# The Role of Th-17 Cells and γδ T-Cells in Modulating the Systemic Inflammatory Response to Severe Burn Injury

**DOI:** 10.3390/ijms18040758

**Published:** 2017-04-03

**Authors:** Albert Kim, Thomas Lang, Meilang Xue, Aruna Wijewardana, Chris Jackson, John Vandervord

**Affiliations:** 1 Severe Burns Unit, Royal North Shore Hospital, St Leonards NSW 2065, Australia; thomas.lang@sydney.edu.au (T.L.); wijewardanaaruna@yahoo.com (A.W.); johnvandervord@bigpond.com (J.V.); 2 Sutton Arthritis Research Laboratory, Institute of Bone and Joint Research, University of Sydney, Sydney NSW 2006, Australia; meilang.xue@sydney.edu.au (M.X.); chris.jackson@sydney.edu.au (C.J.)

**Keywords:** burns, inflammation, systemic inflammatory response, γδ T-cells, cytokines

## Abstract

Burns are a global public health problem, accounting for an estimated 265,000 deaths annually. Inflammation is essential in supplying the growth factors, cytokines and chemokines needed to recruit T-cells and myeloid cells to the site of a burn injury for wound healing. However, major burns generate a marked pathophysiological inflammatory response through a widespread release of abundant pro-inflammatory mediators that predispose patients to a systemic inflammatory response syndrome, sepsis and multi-organ failure. Recently, there has been promising investigation into the role of γδ T-cells and Th-17 cells in the regulation and propagation of this inflammatory response. This study reviews the current literature on the post-burn immune response.

## 1. Introduction

Burns are a global public health problem, accounting for an estimated 300,000 deaths annually [1]. The high incidence of morbidity and mortality associated with major burns is linked to the post-burn inflammatory response, which predisposes patients to the development of a systemic inflammatory response syndrome, sepsis and multi-organ failure [2,3,4].

Inflammation is essential in supplying the growth factors, cytokines and chemokines needed to recruit T-cells and myeloid cells to the site of burn injury for wound healing [5]. However, major burns generate a marked pathophysiological inflammatory response through the widespread release of abundant pro-inflammatory mediators which can be detrimental. While the mechanisms behind the regulation and propagation of this inflammatory response remain unclear, there has been an increasing focus on the role of γδ T-cells and T-helper 17 (Th-17) cells [2,3,4]. The following review aims to summarize the current literature on their roles in the post-burn immune response.

## 2. Th-17 Cells

An appropriate T-cell response is essential to the orchestration of a proper immune response. The Th-1/Th-2 paradigm has long been the accepted basis of the adaptive immune response. Th-1 cells primarily release interleukin-2 (IL-2), interferon-γ (IFN-γ) and tumour necrosis factor-β (TNF-β) to prompt a cell-mediated immune response, while Th-2 cells secrete IL-4, IL-5, IL-10 and IL-13 to initiate a humoral immune response. Major trauma including burns has been demonstrated to attenuate the Th-1 response and enhance the Th-2 response, creating an immunosuppressed state that leads to decreased resistance to infection and increased susceptibility to sepsis [6,7,8]. This is in part due to the inhibitory effects of the Th-2 effector cytokine, IL-10, on the release of essential cytokines such as IFN-γ and TNF-α [9]. More recently, however, newer Th-cell subsets such as Th-17 have been shown to play a major role in trauma [6].

**Figure 1:**
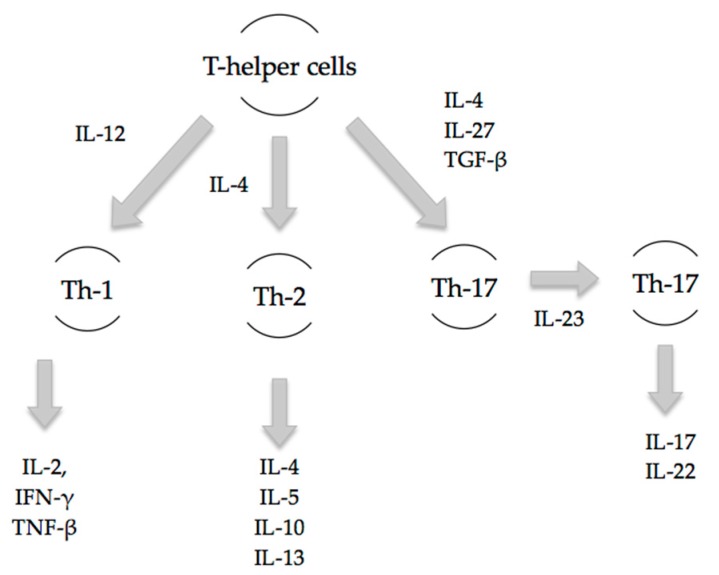
In response to trauma, naïve T-cells differentiate into specific T-cell subsets based on the presence of specific inflammatory mediators. Th-1 cells arise under the influence of IL-12 and provide cell-mediated immunity. Th-2 cells arise under the influence of IL-4 and offer humoral immunity. Th-17 cells arise in the presence of transforming growth factor-β (TGF-β) and IL-23, and produce pro-inflammatory mediators, IL-17 and IL-22 [6].

Th-17 cells are pro-inflammatory and have a distinct lineage to that of Th-1 and Th-2 cells (Figure 1). Early inflammatory mediators, IL-4, IL-27 and TGF-β, regulate and induce the differentiation of naïve T-cells into Th-17 cells [10]. The release of subsequent mediators IL-17A, IL-17F, IL-22 and IL-23 is associated with the mature Th-17 cell phenotype. Local and systemic Th-17 immune responses occur as early as 3 h post-burn injury as evidenced by increases in IL-17 and IL-22 at the burn site, in the systemic circulation, and at distant sites including cardiac and lung tissue [6]. Rani et al. have shown that increases in Th-2 and Th-17 cytokines at the burn site are greater than that of Th-1 cytokines by 10- and 20-fold, respectively. Elevated levels of these cytokines were found to persist at seven days following burn injury [7].

Through the release of IL-17, Th-17 cells propagate inflammation by facilitating neutrophil recruitment and activation. IL-22 helps maintain mucosal immunity by enhancing tight junction formation through ERK MAPK signaling and up-regulating tight junction proteins at mucosal interfaces, strengthening the barrier function of the skin, lung and gut. In this way, Th-17 cells modulate immune and host epithelial homeostasis, particularly at host/environment interfaces [7,10,11]. IL-22 is unique in that it does not act on immune cells but on epithelial cells where it is involved in chemotaxis, tissue repair, antimicrobial peptide expression to prevent invasion by luminal bacteria, and epithelial cell proliferation and differentiation [10]. This role of Th-17 cells is important as major burns induce mucosal gut atrophy and apoptosis, and disruption of gut epithelial cell homeostasis. Gut barrier function is impaired as early as 5 min following major burns, increasing the risk of bacterial translocation and predisposing to sepsis [12]. Th-17 cells have been demonstrated to protect against local and systemic infections from pathogens such as *Bacteroides fragilis*, *Klebsiella pneumonia* and *Candida albicans*, which are common sources of infection following burns [7,10].

It is unclear whether the early robust Th-17 response following burn injury is beneficial or detrimental. While reduced Th-17 responses may lessen bacterial clearance and increase epithelial vulnerability, uncontrolled Th-17 effector activity may facilitate autoimmune disease [10]. Rather than absolute cytokine levels, it is likely that a balanced combination of pro-inflammatory and anti-inflammatory cytokine genotypes resulting in a balanced inflammatory response favors immunocompetence and survival following burn injury [8]. Trauma including burns has systemic consequences, many of which affect the delicate mechanisms that govern Th-17 immunity and may contribute to infection, multi-organ dysfunction, and mortality [10].

## 3. γδ T-Cells

γδ T-cells, a T-cell subset, have a critical role in major burns. Studies that have subjected wild-type and γδ T-cell-deficient mice to thermal injury have demonstrated a causative relationship between γδ T-cell activation, chemokine production and the post-burn inflammatory response [13]. In peripheral lymphoid tissues such as the spleen where αβ T-cells predominate, γδ T-cells are only present at low levels. However, they are the most prevalent T-cell population in epithelial-rich tissues such as the skin and gut [5,14]. They differ from αβ T-cells in that they interact with antigens directly, with no requirement for presentation or processing [15]. The unique characteristics of γδ T-cells, such as their location in barrier tissues, rapid expansion upon activation and ability to independently recognize antigens, make them ideal regulators of local injury and the maintenance of skin homeostasis [13]. Activated γδ T-cells following burn injury predominantly display a Th-17 and Th-2 phenotype [7].

Given their roles in immune surveillance, γδ T-cells are considered more a part of the innate immune system [14]. γδ T-cells in skin survey for signs of epithelial injury or stress and produce chemokines in response to antigens expressed by damaged or stressed keratinocytes. They also play an essential role in the recruitment of inflammatory cells and pro-inflammatory cytokines to the injury site after burn, as evidenced by the significant reduction in cellular filtrate seen in γδ T-cell-deficient mice [5,15]. Studies on mice deficient in γδ T-cells have shown markedly less inflammation, edema and immune cell infiltration with defects in keratinocyte proliferation and tissue re-epithelialization. They also exhibited decreased levels of fibroblast growth factor and platelet-derived growth factor, further suggestive of impaired healing. The addition of γδ T-cells to the culture in in vitro studies subsequently restored normal wound healing [13,15].

Activated γδ T-cells have also been shown to have increased toll-like receptor (TLR) reactivity following burn injury, exhibiting transiently increased expression of TLR2, TLR4 and TLR9. TLRs are cell surface receptors that have a role in innate immunity by recognizing pathogen-associated molecular patterns (PAMP) and activating pro-inflammatory pathways in response to a microbial insult. TLR activation induces the expression of genes that modulate cell migration to the burn site and into the systemic circulation, to help initiate the healing process. The early and transient nature of increased γδ T-cell TLR expression following burn injury is likely protective to prevent detrimental activation [15,16].

In non-pathological conditions, γδ T-cells are the most common T-cell population found in the skin. Their absolute number remains comparable following burn injury, suggesting that those present at the wound site are resident skin cells rather than infiltrating cells [5]. The massive influx of αβ T-cells to the wound following a burn injury appears dependent on the presence of γδ T-cells as decreased chemokine expression and a significantly reduced cellular filtrate are seen in mice lacking γδ T-cells. The percentage of γδ T-cells at the wound site becomes expectedly low following the recruitment of primarily αβ T-cells which remain elevated at seven days post-injury. Thus, although they are not the predominant T-cell population in the burn wound, γδ T-cells appear central to the orchestration of the infiltrating immune response [13,15].

Another important function of γδ T-cells in burn-induced immunopathology is in priming macrophages to produce inflammatory mediators such as TNF-α, IL-6, nitric oxide and PGE2 [13]. High systemic levels of these pro-inflammatory cytokines including TNF-α and IL-6 subsequently induce a cascade of secondary inflammatory mediators, propagating the inflammatory response [13,17]. Macrophages isolated from γδ T-cell-deficient mice at seven days post-burn produced decreased levels of pro-inflammatory cytokines compared with wild-type mice. Macrophage hyperactivity secondary to γδ T-cells—the increased productive capacity of macrophages for inflammatory mediators—has been associated with an increased susceptibility to sepsis after burn injury [17,18]. Increased levels of activated γδ T-cells are seen in the circulation of patients with trauma and sepsis, and blockade of IL-6 activity has been shown to improve the outcome [18].

γδ T-cells also appear to influence myeloid cell recruitment to the site of injury. Macrophages and other myeloid cells such as myeloid-derived suppressor cells (MDSCs) migrate to the wound in the inflammatory phase of wound healing, within 48 h of injury. They release cytokines and growth factors such as platelet-derived growth factor (PDGF) that propagate capillary growth and the formation of granulation tissue seen in the ensuing proliferative phase [13]. MDSCs are thought to play an important role in wound healing via the production of nitric oxide and through promoting angiogenesis to help transition the wound from the inflammatory to proliferative stage of healing. Mice lacking glycoprotein 130 and therefore unable to expand their MDSC population have been shown to display markedly higher mortalities to sepsis associated with increased cytokine production. Survival in these mice improved with reconstitution with MDSCs [19,20]. Thus, MDSCs appear to be immunoprotective, enhancing immune surveillance and innate immune responses through the modulation of macrophage cytokine production. Rani et al. demonstrated a greater increase in macrophages and MDSC recruitment in γδ T-cell-deficient mice compared to wild-type mice at the burn site following injury. This suggests that γδ T-cells at the burn wound may act to suppress myeloid cell influx [19].

Studies have also confirmed that γδ T-cells promote inducible nitric oxide synthase (iNOS) expression and enhance macrophage nitric oxide (NO) production post-burn [21,22]. NO, produced from l-arginine by NOS, is produced in large amounts following burn injury and has been shown to have both therapeutic and deleterious effects on the post-burn immune response. This is supported by studies that have detected supraphysiological levels of NO metabolites in the burn wounds, urine and remote organs of burn patients, including the brain, liver, kidney, spleen and gastrointestinal tract [21,23]. NO is an essential component of wound healing, inducing granulation tissue formation, angiogenesis, epithelial proliferation, collagen synthesis and wound closure [24,25]. Indeed, iNOS-deficient mice have significantly impaired wound healing due to decreased fibroblast proliferation, collagen synthesis and fibroblast-mediated matrix contraction. Macrophage angiogenic activity, another critical component of wound healing, is contingent on iNOS activity. However, in higher concentrations, NO has also been associated with remote organ injury. Chen et al. have implicated iNOS-derived NO in contributing to gastrointestinal dysfunction through increased mucosal permeability, bacterial translocation and villous injury. It is also thought to mediate pulmonary inflammation, edematogenesis and tissue injury which can be reversed with an iNOS inhibitor [23]. Importantly, NO is also immunosuppressive as it depresses the Th-1 cell-mediated immune response and promotes the Th-2 response. Daniel et al. have demonstrated this biphasic response to NO, with low concentrations selectively enhancing the induction of Th-1 cells and higher concentrations suppressing the Th-1 response [21]. A prolonged or unrestricted Th-2 response has been clearly established to be immunosuppressive, increasing the risk of infectious complications and sepsis. This is supported by the presence of higher NO levels in burn patients who become septic [21,26]. Up-regulation of NO following burn injuries is likely due to modulation of macrophages and MDSCs, which are important sources of nitric oxide [13].

Increased levels of the Th-17 effector cytokine, IL-17, likely contribute to the systemic inflammatory response following burn injury. Recent findings have shown a causative relationship between γδ T-cells, IL-17 and survival following sepsis [6]. While IL-17 acts on multiple cell types including neutrophils, fibroblasts, epithelial cells and endothelial cells, its precise role in the early post-burn response remains unclear. Its main role is likely in recruiting immune cells such as neutrophils and monocytes, and propagating inflammation. Indeed, IL-17 deficiencies are associated with defects in neutrophil responses. Early elevation in skin IL-17 following burn injury is thought to be due to activation of the resident γδ T-cell population, though it is also produced by CD8 T-cells, natural killer cells and neutrophils. Sustained elevations in levels of IL-17 may contribute to immune aberrancies promoting sepsis. In a murine model of cecal ligation and puncture as a model for sepsis by Flierl et al., neutralization of elevated IL-17 correlated with decreased plasma levels of pro-inflammatory cytokines TNF-α, IL-1β, and IL-6, decreased bacteremia and increased survival [7,10].

IL-22, the other major immunomodulatory secretory product of Th-17 cells, has also been shown to negatively affect outcomes during sepsis. While IL-22 is generally considered immunoprotective, Weber et al. demonstrated inhibition of IL-22 following cecal ligation and puncture to enhance bacterial clearance, promote phagocyte recruitment, attenuate organ dysfunction, and decrease expression of IL-10, indicating an interdependent relationship between IL-22 and IL-10. This would suggest that adequate levels of IL-22 must be maintained to optimize immune function, whereas higher levels may induce increased expression of IL-10, enhancing bacterial growth and increasing the risk of sepsis [10].

Remote organ injury secondary to the systemic inflammatory response seen in severe burns appears to be primarily mediated by neutrophils. Neutrophil-mediated tissue injury occurs due to the recruitment and activation of sequestrated neutrophils and the subsequent secretion of noxious oxygen free radicals and proteases. As previously mentioned, Th-17 cells propagate neutrophil recruitment and activation through the release of IL-17. γδ T-cells also express chemokine receptors such as MIP-1α and MIP-1β which allow neutrophils to travel along chemotactic gradients to remote organs and cause tissue injury. Toth et al. showed that neutrophil accumulation, tissue chemokine levels and tissue injury were absent in the small intestine of γδ T-cell-deficient mice following burn injury. In wild-type mice, small intestine chemokine levels were elevated and there was a greater population of activated circulating γδ T-cells [14]. This would support a role of γδ T-cells in contributing to distal organ injury following burns.

In summary, γδ T-cells appear to have varied immunopathogenic consequences following burn injury: they contribute to survival as evidenced by the increased mortality seen in γδ T-cell-deficient mice, but are also associated with subsequent immune dysfunction and distal organ injury. Overall, activation of γδ T-cells post-burn is likely to be protective due to their role in immune surveillance, tissue repair, wound healing and in their contribution to host defense against intestinal microflora and the maintenance of barrier function [18]. It can be speculated that the γδ T-cell-mediated tissue injury is likely due to macrophage hyperactivity and the subsequent activation of inflammatory cascades and the generation of other mediators. Similarly, there is growing evidence to support a role for pro-inflammatory Th-17 cells in the immunological response post-burn. Through cytokines IL-17 and IL-22, Th-17 cells facilitate neutrophil recruitment and activation, and prevent bacterial translocation by up-regulating gut barrier function. Novel therapies targeting particular inflammatory mediators to achieve a balanced combination of pro-inflammatory and anti-inflammatory cytokines may be valuable in improving survival following major burn injury. There may also be a role for immunological biomarkers in the early diagnosis of sepsis and prognostication of patients following severe burns.
